# Increasing incidence of uterine carcinosarcoma: A United States Cancer Statistics study

**DOI:** 10.1016/j.gore.2022.100936

**Published:** 2022-02-02

**Authors:** Cheng-I Liao, Michelle Ann Caesar, Danny Lee, Ava Chan, Kathleen M. Darcy, Chunqiao Tian, Daniel S. Kapp, John K. Chan

**Affiliations:** a Department of Obstetrics and Gynecology, Kaohsiung Veterans General Hospital, Kaohsiung, Taiwan; b California Pacific Medical Center Research Institute, San Francisco, CA, USA; c Palo Alto Medical Foundation Research Institute, 795 El Camino Real, Palo Alto, CA 94301, USA; d Gynecologic Cancer Center of Excellence, Gynecologic Surgery and Obstetrics, Uniformed Services University of the Health Sciences, Walter Reed National Military Medical Center, Bethesda, MD, USA; e Department of Radiation Oncology, Stanford University School of Medicine, Stanford, CA, USA; f Division of Gynecologic Oncology, California Pacific / Palo Alto / Sutter Health Research Institute, San Francisco, CA, USA

## Abstract

• The incidence of uterine carcinosarcoma increased over the past 17 years.
• Black women in the South ages 70–74 had the highest incidence.
• Uterine carcinosarcoma increased annually by 2.6% in Hispanic women.

## 1 Introduction

Uterine carcinosarcomas (UCS) is a rare tumor that contains carcinomatous and sarcomatous elements. (Berton-Rigaud et al., 2014) Due to its low incidence, UCS has been understudied. UCS is often diagnosed at an older age; however, a prior study showed that UCS incidence has increased in all age groups. (Matsuo et al., 2018) With respect to race, the Black-White disparity in cancer burden exist, particularly in uterine cancer with high risk histologies. (Long et al., 2013, Abel et al., 2021, DeSantis et al., 2019) A prior study from North American Association of Central Cancer Registries (NAACCR) for 49 states and the District of Columbia showed that there are distinct geographic disparity patterns in uterine cancer incidence. However, few studies have focused on UCS incidence and trends based on age, race, and regional disparities in the United States. (Siegel et al., 2013) Thus, we propose to investigate trends in the incidence of UCS with respect to demographic characteristics.

## 2 Methods

Data on UCS were obtained from the United States Cancer Statistics (USCS) Public Use Database from 2001 to 2017. We included both the old and new classifications, the 2003 World Health Organization (WHO) Classification as mixed epithelial and mesenchymal tumors and the 2014 WHO Classification as carcinosarcoma. In the USCS data, the International Classification of Diseases for Oncology (ICD-O-3, 2000) coding was used. SEER*Stat 8.3.9, Joinpoint regression program 4.8.0.1, and Excel were used to calculate incidence and trends. Since this study focused on the period from 2001 to 2017, age-adjusted incidence was estimated relative to U.S. 2000 standard population. Trends were described using average annual percent change (AAPC) and annual percent change (APC). The study was exempt from IRB approval as it contains de-identified data.

## 3 Results

The incidence of uterine carcinosarcoma in the year 2017 was 1.36/100,000 women (Table 1). There were significant increases in incidence in those 55–79 years old (yo), with the largest average annual percent change (AAPC) of 2.96% in the 70–74 yo group (*P* = 0.03) (Table 2). Black women had the highest incidence of UCS in 2017 at 3.16/100,000, followed by Hispanics at 1.27/100,000, 1.11/100,000 in Whites, and 1.05/100,000 in Asians. The largest AAPC was seen in Hispanics at 2.61%, followed by 1.88% in Blacks, and 1.01% in Whites (*P* < 0.001). Furthermore, increases occurred in all U.S. regions, with the largest AAPC in the South at 1.89% (*P* < 0.001). Intersection analyses demonstrated that the highest incidence in 2017 was in Black women, 70–74 yo, in the South at 22.05/100,000 (Table 2). In 2001, this intersection group had an incidence of 15.26/100,000 and has increased annually by 2.19% over the past 17 years (*P* = 0.011) and the highest AAPC was in White women, 70–74 yo, in the Midwest (*P* < 0.001).

**Table 1:** Age-Adjusted Incidences and Trends of Uterine Carcinosarcoma by Race/Ethnicity, Region, and Stage in USCS Public Use Databases from 2001 to 2017^†^

	AAI	Trend 1		Trend 2		Trend 3		2001–2017
	2001 to 2017	Years	APC (95% CI)	Year	APC (95% CI)	Year	APC (95% CI)	AAPC (95% CI)
**Uterine Carcinosarcoma**	1.02 to 1.36	2001–2017	1.51* (1.22 to 1.80)					1.51* (1.22 to 1.80)
**Race/Ethnicity**								
NHW	0.91 to 1.11	2001–2017	1.01*(0.66 to 1.37)					1.01*(0.66 to 1.37)
NHB	2.17 to 3.16	2001–2017	1.88* (1.28 to 2.48)					1.88* (1.28 to 2.48)
Hispanic	0.85 to 1.27	2001–2017	2.61* (1.72 to 3.50)					2.61* (1.72 to 3.50)
NHAPI	0.78 to 1.05	2001–2017	1.39 (-0.13 to 2.93)					1.39 (-0.13 to 2.93)
**Region**								
Northeast	1.28 to 1.47	2001–2017	1.04* (0.34 to 1.74)					1.04* (0.34 to 1.74)
Midwest	0.97 to 1.36	2001–2017	1.43* (0.69 to 2.18)					1.43* (0.69 to 2.18)
South	0.97 to 1.39	2001–2017	1.89* (1.50 to 2.27)					1.89* (1.50 to 2.27)
West	0.91 to 1.23	2001–2017	1.80* (1.38 to 2.23)					1.80* (1.38 to 2.23)
**Stage**								
Local	0.39 to 0.58	2001–2015	1.14* 0.40 to 1.88)	2015–2017	10.28 (-2.59 to 24.85)			2.24* (0.71 to 3.79)
Regional	0.32 to 0.41	2001–2014	2.87* (1.93 to 3.82)	2014–2017	−6.17 (-12.95 to 1.14)			1.11 (-0.33 to 2.57)
Distant	0.23 to 0.32	2001–2017	1.89* (1.40 to 2.38)					1.89* (1.40 to 2.38)
Unknown	0.08 to 0.06	2001–2015	−4.96* (-6.54 to −3.35)	2015–2017	18.18 (-18.33 to 71.01)			−2.33 (-6.50 to 2.02)

† Trends based on incidence were analyzed using the Joinpoint Regression Program, version 4.8.0.1, allowing up to 3 joinpoints. AAPC is a summary measure of the trend over a pre-specified fixed interval. It was computed as a weighted average of the APC from the joinpoint model, with the weights equal to the length the APC interval.

* The APC or AAPC is significantly different from zero (*p* < 0.05).

**Table 2:** Age-Specified Incidences and Trends of Uterine Carcinosarcoma in USCS Public Use Databases from 2001 to 2017^†^

	ASI	Trend 1		Trend 2		Trend 3		2001–2017
	2001 to 2017	Years	APC (95% CI)	Year	APC (95% CI)	Year	APC (95% CI)	AAPC (95% CI)
**Uterine Carcinosarcoma**								
**Age Group**								
40–44	0.26 to 0.24	2001–2017	1.07 (-0.21 to 2.37)					1.07 (-0.21 to 2.37)
45–49	0.44 to 0.39	2001–2017	0.60 (-1.07 to 2.28)					0.60 (-1.07 to 2.28)
50–54	0.85 to 1.21	2001–2004	16.33* (5.65 to 28.08)	2004–2007	−7.27 (-21.34 to 9.31)	2007–2017	1.74* (0.45 to 3.05)	2.53 (-0.67 to 5.83)
55–59	1.88 to 3.00	2001–2005	7.51* (0.71 to 14.76)	2005–2017	1.04* (0.11 to 1.98)			2.62* (1.00 to 4.27)
60–64	3.46 to 5.20	2001–2017	2.53* (1.91 to 3.16)					2.53* (1.91 to 3.16)
65–69	4.84 to 6.84	2001–2017	2.08* (1.50 to 2.66)					2.08* (1.50 to 2.66)
70–74	5.08 to 8.08	2001–2011	4.01* (2.70 to 5.33)	2011–2014	−3.80 (-16.55 to 10.90)	2014–2017	6.56 (-0.01 to 13.56)	2.96* (0.30 to 5.70)
75–79	5.83 to 6.89	2001–2017	1.08* (0.38 to 1.79)					1.08* (0.38 to 1.79)
80–84	6.23 to 7.07	2001–2017	0.30 (-0.42 to 1.02)					0.30 (-0.42 to 1.02)
85+	5.20 to 4.22	2001–2017	−1.95* (-3.01 to −0.87)					−1.95* (-3.01 to −0.87)
**Intersection Groups**								
Black, 70–74, South	15.26 to 22.05	2001–2017	2.19* (0.58 to 3.82)					2.19* (0.58 to 3.82)
White, 70–74, Midwest	2.94 to 7.58	2001–2017	3.80* (2.13 to 5.49)					3.80* (2.13 to 5.49)

† Trends based on incidence were analyzed using the Joinpoint Regression Program, version 4.8.0.1, allowing up to 3 joinpoints. AAPC is a summary measure of the trend over a pre-specified fixed interval. It was computed as a weighted average of the APC from the joinpoint model, with the weights equal to the length the APC interval.

* The APC or AAPC is significantly different from zero (*p* < 0.05).

## 4 Discussion

### 4.1 Overall findings

In our study, we found that uterine carcinosarcoma increased over the past 17 years. Additionally, we found that UCS has been increasing in young women.

### 4.2 Age

UCS is diagnosed at older age, which could be due to changing demographics and the increase in older women. (Kontis et al., 2017) Early diagnosis remains a challenge as most symptoms are nonspecific. Most cases are diagnosed as incidental findings after hysterectomy or morcellation of fibroids. UCS are usually diseases found in elderly women. Our findings of the increasing UCS incidence may be due to increasing numbers of older females in the United States.

However, we also found uterine carcinosarcoma incidence increasing among younger ages as well. Matsuo et al. found an increasing proportion of uterine carcinosarcoma within endometrial cancer in females younger than 60, which the authors attributed to other factors at play. (Matsuo et al., 2018) One factor Matsuo et al. suggested was obesity, which has been increasing among the US population. (Matsuo et al., 2018) More than one third of the adult population was estimated to be obese (37.7%) in 2013–2014. (Ogden et al., 2017) Obesity increases the risk of endometrial cancer and increases the epithelial-mesenchymal transition in endometrial tumors, which may further form uterine carcinosarcoma. (Matsuo et al., 2018)

### 4.3 Race

Our findings are consistent with Rojas *et al*, which found increases in UCS incidence from 3.1% to 14.8% in Black women and 1.5% to 6.7% in White women ages 40 to 90 years old. (Rojas et al., 2020) Similar to our findings, Matsuo *et al* found an increase in UCS incidence in Black women, however, this study found a decrease in UCS incidence in White women. (Matsuo et al., 2018) A prior study evaluating the association between UCS and race found that Black women have an increased risk of developing UCS compared to other racial and ethnic groups. (Brooks et al., 2004) One study found that lower socioeconomic status in Blacks was associated with treatments delays, and upregulated HER2 in tumors from Blacks was associated with treatment resistance and poor survival. (Long et al., 2013)

There are several limitations to this study. Diagnosis could not be confirmed without records to perform pathology or medical oncology reviews. As such we could not attribute increasing incidence to improved pathologic assessment. Additionally, the covariates and endpoints available in the USCS Public Use Database are limited. However, the USCS Public Use Database comprises over 31 million cancer cases, which represents over 99% of the U.S. population. (U.S. Cancer Statistics Public Use Databases | CDC, 2021) Furthermore, this is one of the few studies that have used the USCS Public Use Database to investigate trends in incidence of UCS and the intersectionality of demographic factors.

## 5 Conclusion

The incidence of uterine carcinosarcomas is increasing, most notably in older Black women, and justify research and investment in prevention and early detection.

Author Contribution

All authors made important contributions to this study including conception (CL, MAC, DL, AC, KMD, CT, DSK, JKC), design (CL, MAC, DL, AC, KMD, CT, DSK, JKC), data acquisition (CL, MAC, DL, AC, KMD, CT, DSK, JKC), data analysis (CL), and editing (CL, MAC, DL, AC, KMD, CT, DSK, JKC).

## Declaration of Competing Interest

The authors declare the following financial interests/personal relationships which may be considered as potential competing interests: [Dr. John K. Chan has received non-specific funding from Denise Cobb Hale Chair, Angela Wang Johnson and the Fisher Family Fund; consulted for AstraZeneca, Glaxosmithkline, and Myriad; received payment or honoraria from AstraZeneca, Clovis, Eisai, Glaxosmithkline, Merck, and Roche; and participated on a Data Safety Monitoring Board or Advisory Board for AbbVie, AstraZeneca, Clovis, Eisai, Glaxosmithkline, Immunogen, Myriad, Roche, and Seagen. The other co-authors do not have any competing interests].
